# Supplementary material for: Substantial trace metal input from the 2022 Hunga Tonga-Hunga Ha’apai eruption into the South Pacific
Source: Nat Commun. 2024 Oct 18;15:8986. doi: 10.1038/s41467-024-52904-3 (PMC11487076; doi:10.1038/s41467-024-52904-3)
Supplement: Supplementary file 1 — Supplementary Information [file 41467_2024_52904_MOESM1_ESM.pdf]

Supplementary Information

Substantial trace metal input from the 2022 Hunga Tonga-Hunga Ha'apai eruption into the South Pacific

Zhouling Zhang[1*], Antao Xu[1†], Ed Hathorne[1], Marcus Gutjahr[1], Thomas J. Browning[1], Kathleen J. Gosnell[1], Te Liu[1#], Zvi Steiner[1], Rainer Kiko[1,2], Zhongwei Yuan[1,3], Haoran Liu[1,3], Eric P. Achterberg[1], Martin Frank[1]

[1] GEOMAR Helmholtz Centre for Ocean Research Kiel, Kiel, Germany

[2] Faculty of Mathematics and Natural Sciences, Kiel University, Kiel, Germany

[3] State Key Laboratory of Marine Environmental Science and College of Ocean and Earth Sciences, Xiamen University, Xiamen, China

[†] Now at Institute of Environmental Physics, Heidelberg University, Heidelberg, Germany

[#] Now at School of Ocean and Earth Science, University of Southampton, Southampton, UK

* Corresponding author: zzhang@geomar.de

Content: Supplementary Figures 1-9

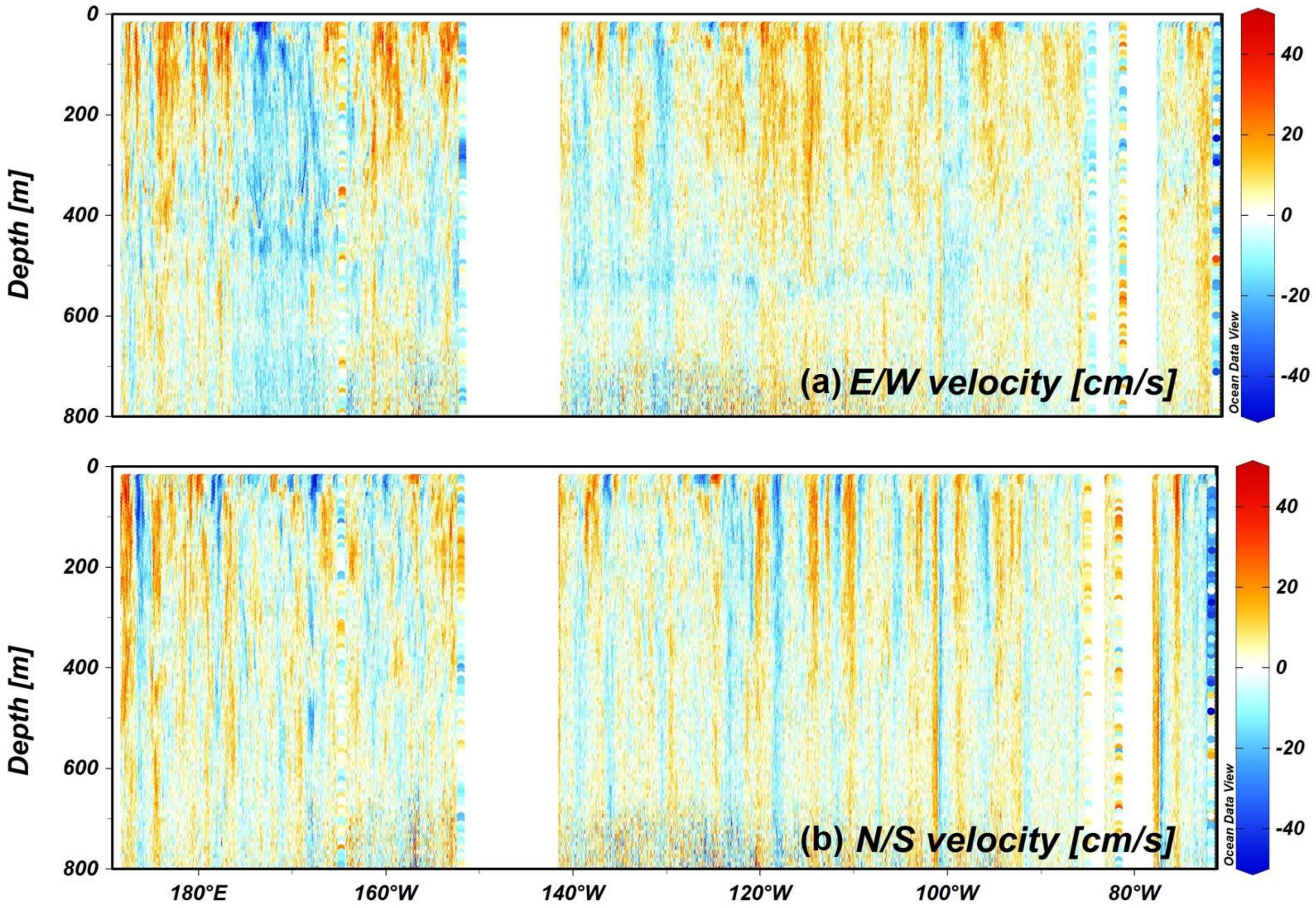

Supplementary Figure 1. **Upper-ocean acoustic Doppler current profiler (75kHz-ADCP) underway data along the cruise track.** (a) The velocities in the East-West (E/W) direction (positive: eastward; negative: westward); (b) The velocities in the North-South (N/S) direction (positive: northward; negative: southward).

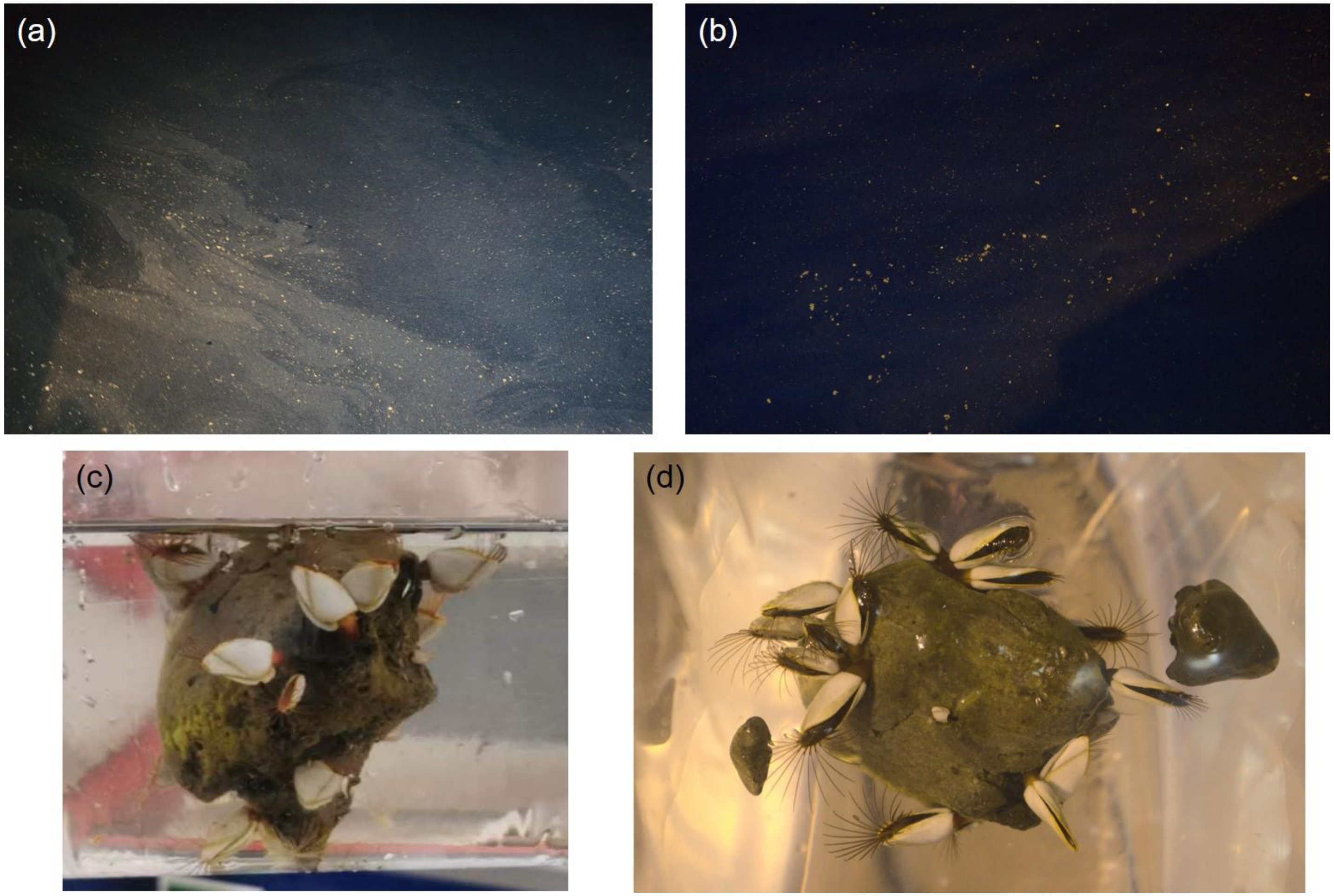

Supplementary Figure 2. **Additional photos of floating pumice observed during the cruise before crossing the Tonga-Kermadec Ridge on 30 March 2022.** (a,b) Floating pumice observed from the vessel. (c,d) Individual pumice that were collected. Photos credit: Maria de los Angeles Amenabar and Te Liu.

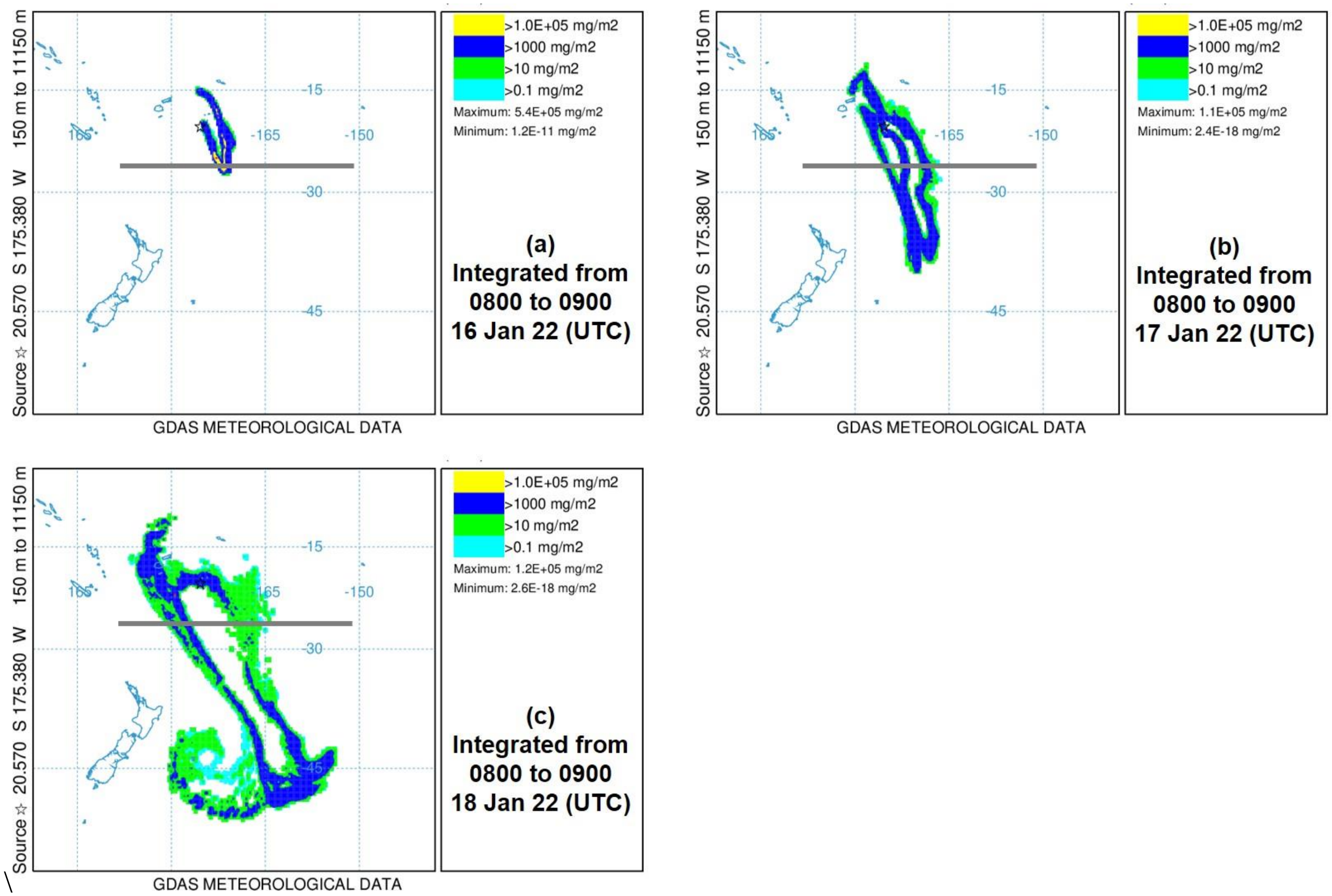

Supplementary Figure 3. **Volcanic ash mass loading (mg/m^2) between 0 m and 18000 m after the Hunga_Tonga-Hunga_Ha'apai (HTHH) volcanic eruption based on the NOAA HYSPLIT volcanic ash dispersion model [1].** (a) 24 hours, (b) 48 hours, and (c) 72 hours after eruption. The grey line in all subfigures represents our western transect (Sta. 33 to 44). Model details and parameterization are given in the Methods section.

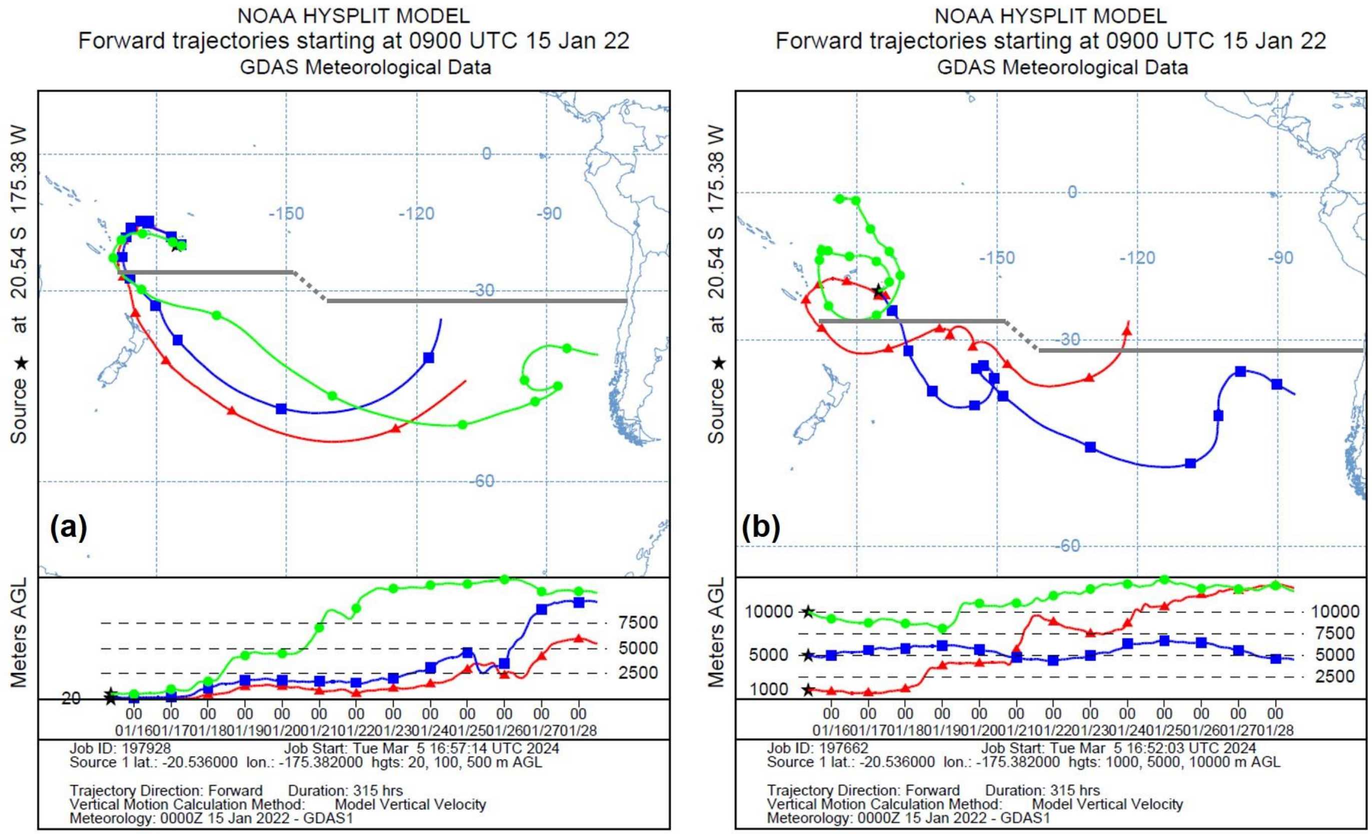

Supplementary Figure 4. **Forward atmospheric trajectories for 315 hours after the HTHH eruption based on the NOAA HYSPLIT trajectory model** [1]**.** (a) Trajectories at 20 (red), 100 (blue), and 500 (green) meters above model ground level. (b) Trajectories at 1000 (red), 5000 (blue), and 10000 (green) meters above model ground level. The trajectories are labelled every 24 hours with a symbol. The grey line represents our full transect. Model details and parameterization are given in the Methods section.

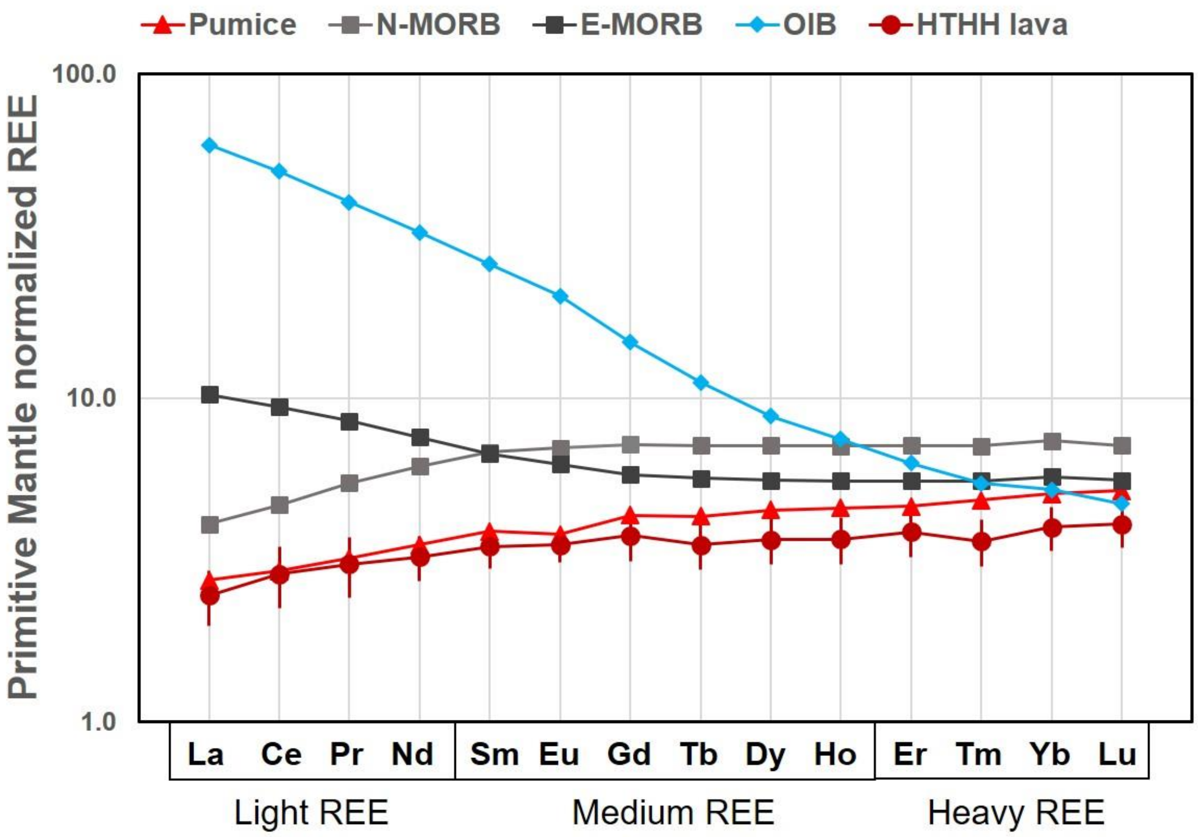

Supplementary Figure 5. **Rare Earth Element (REE) patterns of various types of basalts normalized to Primitive Mantle.** REE data for mid-ocean ridge basalts (MORB) and ocean island basalts (OIB) are from Sun and McDonough [2], where those values are based on a literature review and internal consistency of elemental ratios. N-MORB represents normal MORB and E-MORB represents enriched MORB. REE data for Hunga Tonga-Hunga Ha'apai (HTHH) lava are from Ewart et al. [3], where the error bar represents the standard deviation of five lava samples.

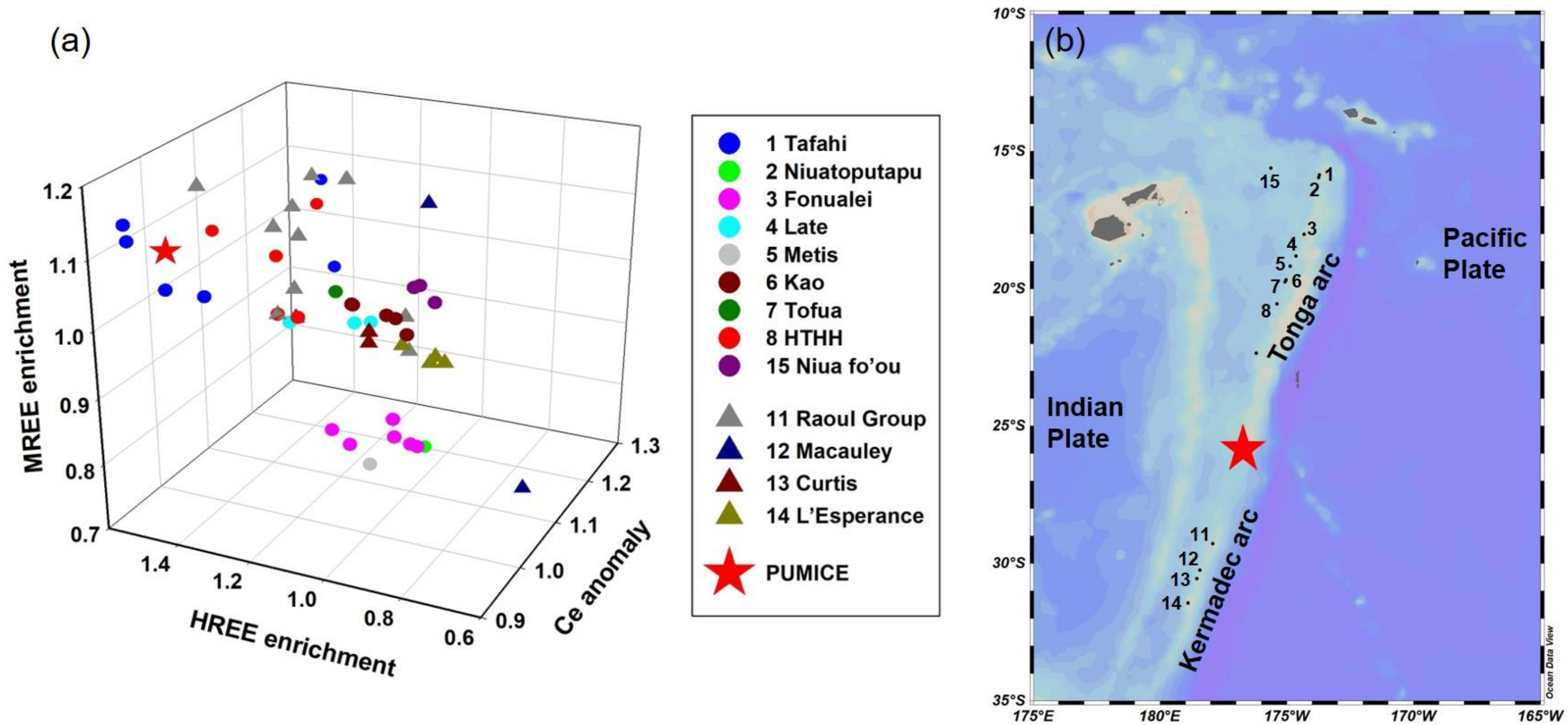

Supplementary Figure 6. **Primitive mantle normalized Rare Earth Element (REE) patterns of lavas from different Tonga-Kermadec islands.** (a) Primitive mantle normalized Ce anomaly (x-axis; calculated as 2×Ce/(La+Pr)), heavy REE (HREE) enrichment (y-axis; calculated as (Yb+Lu)/(Pr+Nd)), and medium REE (MREE) enrichment (z-axis; calculated as 2*(Gd+Tb+Dy)/(La+Pr+Nd+Er+Yb+Lu)) of Tonga lavas (in circles), Kermadec lavas (in triangles), and the Pumice (in star). REE data for the primitive mantle are taken from Hofmann [4]. REE data for the lavas are from Ewart et al. [3] (b) Map of the

Tonga-Kermadec Arc showing the locations of all the islands/volcanos listed in subfigure a. The red star shows where we collected the Pumice. The REE characteristics of the Pumice are similar to lavas from the Hunga Tonga-Hunga Ha'apai (HTHH), Tafahi, and Raoul islands. The last known volcanic eruption of Raoul Island occurred in 2006 (https://volcano.si.edu/volcano.cfm?vn=242030), and no eruptions have been reported for the Tafahi Island (https://volcano.si.edu/volcano.cfm?vn=243101). The Pumice is most likely derived from the eruptions of HTHH volcano.

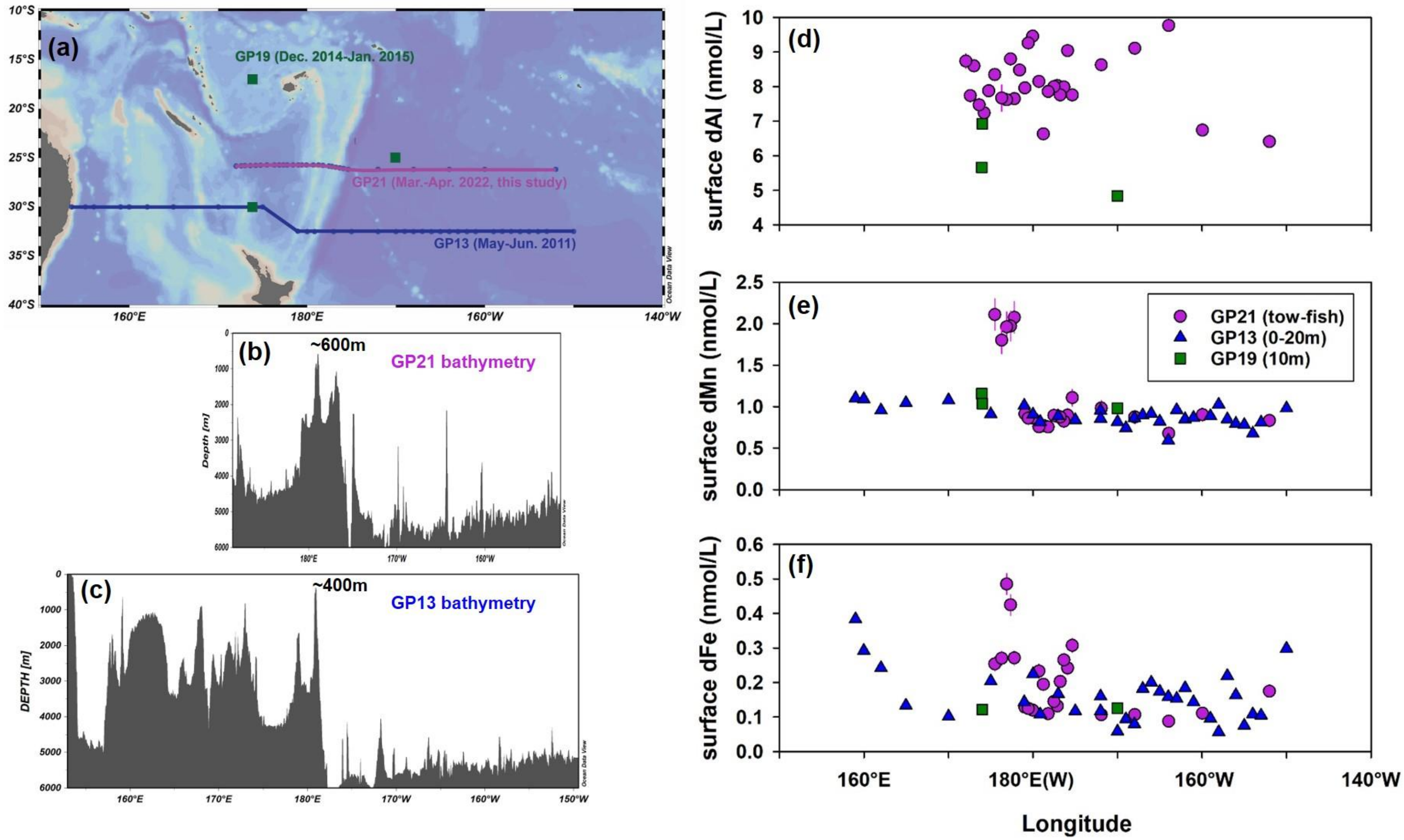

Supplementary Figure 7. **A comparison of trace metal concentrations in the surface water of the western South Pacific Gyre (150°E to 140°W) between three GEOTRACES cruises, GP13, GP19 and GP21 (this study).** (a) The map

shows the sampling locations used in the comparison from the aforementioned cruises. (b-c) shows the bathymetry of the western section of GP21 and GP13. The Tonga-Kermedec ridge that both cruises crossed showed similar depth (~400-600m). (d-f) shows the comparison of surface dissolved aluminium (dAl), manganese (dMn) and iron (dFe) concentrations. Data for GP13 is from the GEOTRACES intermediate data product 2021 (version 2) [5]. Data for GP19 is from Zheng et al. [6] and the GEOTRACES intermediate data product 2021 (version 2) [5]. The error bars of dAl, dMn, and dFe from our cruise represent 1SD of repeated measurements of these trace metals, respectively.

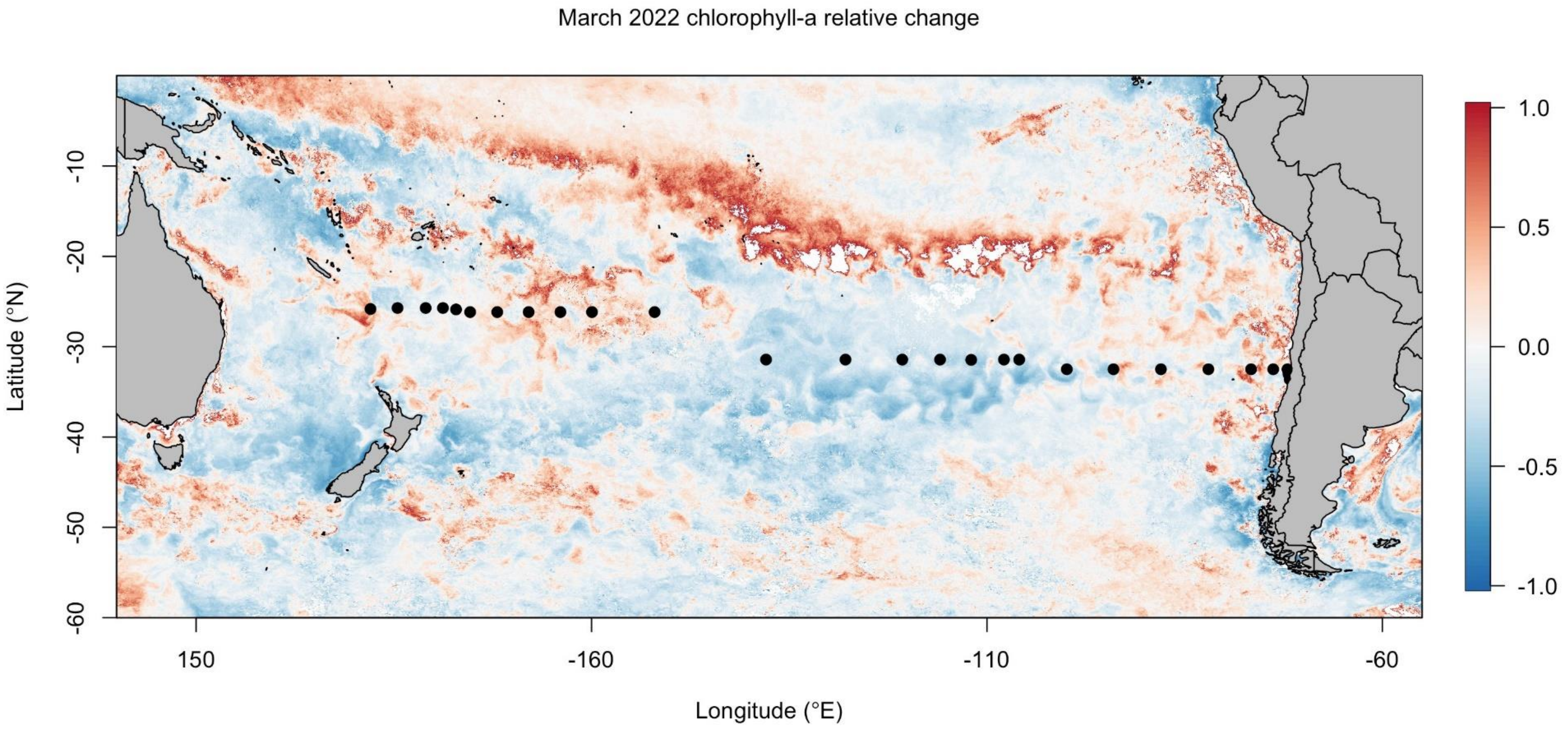

Supplementary Figure 8. **Map of satellite-derived chlorophyll-a anomalies showing the relative change in chlorophyll-a concentrations in March 2022 compared to the March average for the period 2002 to 2023 in the South Pacific Ocean.** The satellite product employed was Aqua-MODIS Chlorophyll-a from NASA (https://oceancolor.gsfc.nasa.gov/) [7].

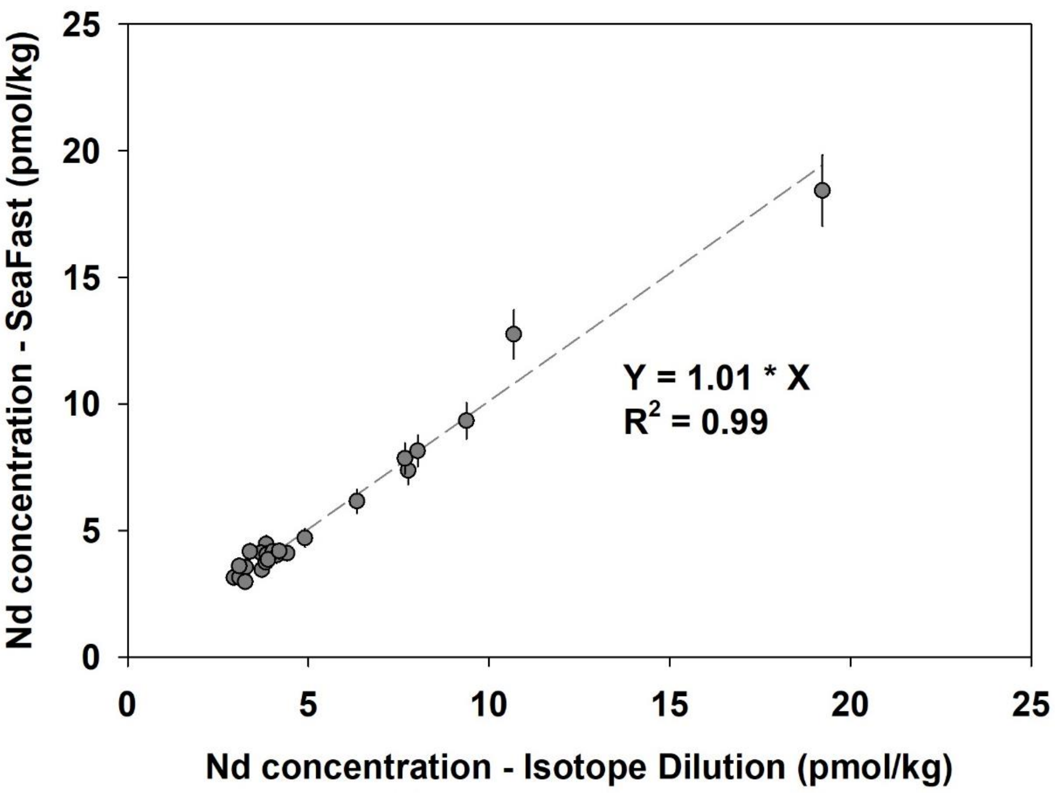

Supplementary Figure 9. **Comparison of Nd concentrations measured using SeaFast and the isotope dilution method in this study.** The error bar represents the external 2SD of the measurement using SeaFast. The external 2SD of the measurement using Isotope Dilution are smaller than symbol size.

References

1. Stein, A. F. *et al.* NOAA's HYSPLIT Atmospheric Transport and Dispersion Modeling System. *Bull Am Meteorol Soc* **96**, 2059–2077 (2015).
2. Sun, S. -s. & McDonough, W. F. Chemical and isotopic systematics of oceanic basalts: implications for mantle composition and processes. *Geological Society, London, Special Publications* **42**, 313–345 (1989).
3. Ewart, A., Collerson, K. D., Regelous, M., Wendt, J. I. & Niu, Y. Geochemical Evolution within the Tonga-Kermadec-Lau Arc-Back-arc Systems: the Role of Varying Mantle Wedge Composition in Space and Time. *Journal of Petrology* **39**, 331–368 (1998).
4. Hofmann, A. W. Chemical differentiation of the Earth: the relationship between mantle, continental crust, and oceanic crust. *Earth Planet Sci Lett* **90**, 297–314 (1988).
5. GEOTRACES Intermediate Data Product Group. *The GEOTRACES Intermediate Data Product 2021v2 (IDP2021v2)*. (2023) doi:10.5285/ff46f034-f47c-05f9-e053-6c86abc0dc7e.
6. Zheng, L., Minami, T., Takano, S. & Sohrin, Y. Distributions of aluminum, manganese, cobalt, and lead in the western South Pacific: Interplay between the South and North Pacific. *Geochim Cosmochim Acta* **338**, 105–120 (2022).
7. NASA Ocean Biology Processing Group. Moderate-resolution imaging spectroradiometer (MODIS) Aqua L3 chlorophyll data 2022 reprocessing [Dataset]. *OB.DAAC.* Retrieved from https://oceancolor.gsfc.nasa.gov/l3/order/. (2022)
