# Supplementary material for: Substantial trace metal input from the 2022 Hunga Tonga-Hunga Ha’apai eruption into the South Pacific
Source: Nat Commun. 2024 Oct 18;15:8986. doi: 10.1038/s41467-024-52904-3 (PMC11487076; doi:10.1038/s41467-024-52904-3)
Supplement: Supplementary file 2 — Description of Additional Supplementary Files [file 41467_2024_52904_MOESM2_ESM.pdf]

Description of Additional Supplementary Files

File Name: Supplementary Data 1
Description: This dataset contains dissolved radiogenic neodymium (Nd) isotopes, rare earth element (REE) and trace metal (TM) concentrations of surface water and euphotic layer chlorophyll-a inventories in the South Pacific Ocean collected during the Pacific GEOTRACES GP21 expedition.
