# Supplementary material for: Substantial trace metal input from the 2022 Hunga Tonga-Hunga Ha’apai eruption into the South Pacific
Source: Nat Commun. 2024 Oct 18;15:8986. doi: 10.1038/s41467-024-52904-3 (PMC11487076; doi:10.1038/s41467-024-52904-3)
Supplement: Supplementary file 4 — Reporting Summary [file 41467_2024_52904_MOESM4_ESM.pdf]

nature portfolio

Corresponding author(s): Zhouling Zhang

Last updated by author(s): Sep 22, 2024

Reporting Summary

Nature Portfolio wishes to improve the reproducibility of the work that we publish. This form provides structure for consistency and transparency in reporting. For further information on Nature Portfolio policies, see our Editorial Policies and the Editorial Policy Checklist.

Statistics

For all statistical analyses, confirm that the following items are present in the figure legend, table legend, main text, or Methods section.

n/a	Confirmed	
☐	☒	The exact sample size (*n*) for each experimental group/condition, given as a discrete number and unit of measurement
☐	☒	A statement on whether measurements were taken from distinct samples or whether the same sample was measured repeatedly
☐	☒	The statistical test(s) used AND whether they are one- or two-sided<br>*Only common tests should be described solely by name; describe more complex techniques in the Methods section.*
☒	☐	A description of all covariates tested
☒	☐	A description of any assumptions or corrections, such as tests of normality and adjustment for multiple comparisons
☐	☒	A full description of the statistical parameters including central tendency (e.g. means) or other basic estimates (e.g. regression coefficient) AND variation (e.g. standard deviation) or associated estimates of uncertainty (e.g. confidence intervals)
☐	☒	For null hypothesis testing, the test statistic (e.g. F, t, r) with confidence intervals, effect sizes, degrees of freedom and P value noted<br>*Give P values as exact values whenever suitable.*
☒	☐	For Bayesian analysis, information on the choice of priors and Markov chain Monte Carlo settings
☒	☐	For hierarchical and complex designs, identification of the appropriate level for tests and full reporting of outcomes
☒	☐	Estimates of effect sizes (e.g. Cohen's d, Pearson's r), indicating how they were calculated

Our web collection on statistics for biologists contains articles on many of the points above.

Software and code

Policy information about availability of computer code

Data collection	MC-ICP-MS: NEPTUNE PLUS software "Tune" (Thermo Scientific)<br>ICP-MS: ELEMENT 2/XR software version 3.1 (Thermo Scientific)<br>High performance liquid chromatography: Chromeleon version 7.0 (Thermo Fisher Scientific).
Data analysis	Statistics and other calculations were conducted using SigmaPlot version 14.0 and Ocean Data View 5.6.3

For manuscripts utilizing custom algorithms or software that are central to the research but not yet described in published literature, software must be made available to editors and reviewers. We strongly encourage code deposition in a community repository (e.g. GitHub). See the Nature Portfolio guidelines for submitting code & software for further information.

Data

Policy information about availability of data

All manuscripts must include a data availability statement. This statement should provide the following information, where applicable:

- Accession codes, unique identifiers, or web links for publicly available datasets
- A description of any restrictions on data availability
- For clinical datasets or third party data, please ensure that the statement adheres to our policy

Source data are provided with this paper. Experimental data are provided in the Supplementary Data 1, which contains the data used to produce Figure 2-6.

Research involving human participants, their data, or biological material

Policy information about studies with human participants or human data. See also policy information about sex, gender (identity/presentation), and sexual orientation and race, ethnicity and racism.

Reporting on sex and gender	*Use the terms sex (biological attribute) and gender (shaped by social and cultural circumstances) carefully in order to avoid confusing both terms. Indicate if findings apply to only one sex or gender; describe whether sex and gender were considered in study design; whether sex and/or gender was determined based on self-reporting or assigned and methods used. Provide in the source data disaggregated sex and gender data, where this information has been collected, and if consent has been obtained for sharing of individual-level data; provide overall numbers in this Reporting Summary. Please state if this information has not been collected. Report sex- and gender-based analyses where performed, justify reasons for lack of sex- and gender-based analysis.*
Reporting on race, ethnicity, or other socially relevant groupings	*Please specify the socially constructed or socially relevant categorization variable(s) used in your manuscript and explain why they were used. Please note that such variables should not be used as proxies for other socially constructed/relevant variables (for example, race or ethnicity should not be used as a proxy for socioeconomic status). Provide clear definitions of the relevant terms used, how they were provided (by the participants/respondents, the researchers, or third parties), and the method(s) used to classify people into the different categories (e.g. self-report, census or administrative data, social media data, etc.) Please provide details about how you controlled for confounding variables in your analyses.*
Population characteristics	*Describe the covariate-relevant population characteristics of the human research participants (e.g. age, genotypic information, past and current diagnosis and treatment categories). If you filled out the behavioural & social sciences study design questions and have nothing to add here, write "See above."*
Recruitment	*Describe how participants were recruited. Outline any potential self-selection bias or other biases that may be present and how these are likely to impact results.*
Ethics oversight	*Identify the organization(s) that approved the study protocol.*

Note that full information on the approval of the study protocol must also be provided in the manuscript.

Field-specific reporting

Please select the one below that is the best fit for your research. If you are not sure, read the appropriate sections before making your selection.

☐ Life sciences ☐ Behavioural & social sciences ☒ Ecological, evolutionary & environmental sciences

For a reference copy of the document with all sections, see nature.com/documents/nr-reporting-summary-flat.pdf

Ecological, evolutionary & environmental sciences study design

All studies must disclose on these points even when the disclosure is negative.

Study description	Field sampling and experiments were conducted aboard the German research vessel SONNE (SO289) during the GEOTRACES GP21 trans-Pacific (Chile-New Caledonia) expedition, which took place between 23 February and 4 April 2022. Surface seawater samples were collected for dissolved radiogenic neodymium (Nd) isotopes, rare earth element (REE) and trace metal (TM) concentrations, and chlorophyll-a concentrations in the South Pacific Ocean to investigate the external input of trace metals to the surface layer and the biological response to this input.
Research sample	Natural trace metal concentrations in surface seawater collected during the research cruise and in pumice samples floating in surface water near the Tonga-Kermedec Arc.
Sampling strategy	20-40 L of surface seawater was collected for Nd isotopes and REE concentrations either from the Niskin bottles attached to a stainless steel CTD rosette or from an underwater trace metal-cleaned tow-fish. TM samples for dissolved manganese (dMn), dissolved iron (dFe), and dissolved aluminum (dAl) were collected from the tow-fish. Seawater samples for phytoplankton pigment analysis were collected in 10 L opaque carboys at six depths throughout the euphotic zone. The depths were identified by the photosynthetically active radiation (PAR) intensity using a PAR sensor and an in vivo fluorescence sensor attached to the CTD (i.e., 100%, 50%, 25%, 10%, 1%, 0.1% surface PAR). The pumice samples were collected using a bucket.
Data collection	Samples were collected by Z. Zhang, A. Xu, K. Gosnell, T. Liu, Z. Steiner, T. Browning, Z. Yuan and H. Liu during the research cruise. The persons who carried out the measurements and the instruments used for data collection were:<br>Radiogenic Nd isotopes: Z. Zhang, Neptune Plus Multicollector-Inductively Coupled Plasma Mass Spectrometer (MC-ICP-MS)<br>Dissolved rare earth element concentrations: Z. Zhang; Thermo Element XR ICP-MS<br>dissolved Fe and Mn concentrations: K. Gosnell; Thermo Element XR ICP-MS<br>Dissolved Al concentrations: T. Liu; Carey Eclipse fluorimeter<br>Chlorophyll-a concentrations: T. Browning, Z. Yuan and H. Liu; high-performance liquid chromatography (HPLC) (Dionex UltiMate 3000 LC system, Thermo Scientific)

Timing and spatial scale	Samples were collected between February 23 and April 4, 2022 at 26-32°S from 70°W to 170°E across the subtropical South Pacific.
Data exclusions	Surface water trace metal data and euphotic layer chlorophyll a data east of 90°W are excluded as they are subject to input from the Chilean coast and are not relevant to our discussion of external input in the western South Pacific.
Reproducibility	Surface and deep seawater samples were selected from a random station for repeated measurements of rare earth elements, which showed great reproducibility during the measurement.
Randomization	Sampling bottles were filled at random.
Blinding	All analytical measurements were assigned independent sample IDs and the analyst was therefore blinded to sample location and/or depth.

Did the study involve field work? ☒ Yes ☐ No

Field work, collection and transport

Field conditions	The weather in the study area has been kind and we have not lost any station time as a result of poor weather.
Location	Samples were collected at 26-32°S from 70°W to 170°E across the subtropical South Pacific. Samples were all collected from the near-sea surface (~3-5 m depth), except for the Chlorophyll-a concentrations, which were collected from the euphotic layer.
Access & import/export	Samples were acquired and transported back to Germany adhering to sampling and import/export policies of GEOMAR (Kiel, Germany).
Disturbance	All chemicals and seawater exposed to chemicals were transported back to Germany for disposal.

Reporting for specific materials, systems and methods

We require information from authors about some types of materials, experimental systems and methods used in many studies. Here, indicate whether each material, system or method listed is relevant to your study. If you are not sure if a list item applies to your research, read the appropriate section before selecting a response.

Materials & experimental systems

n/a	Involved in the study
☒	☐ Antibodies
☒	☐ Eukaryotic cell lines
☒	☐ Palaeontology and archaeology
☒	☐ Animals and other organisms
☒	☐ Clinical data
☒	☐ Dual use research of concern
☒	☐ Plants

Methods

n/a	Involved in the study
☒	☐ ChIP-seq
☒	☐ Flow cytometry
☒	☐ MRI-based neuroimaging

Plants

Seed stocks	*Report on the source of all seed stocks or other plant material used. If applicable, state the seed stock centre and catalogue number. If plant specimens were collected from the field, describe the collection location, date and sampling procedures.*
Novel plant genotypes	*Describe the methods by which all novel plant genotypes were produced. This includes those generated by transgenic approaches, gene editing, chemical/radiation-based mutagenesis and hybridization. For transgenic lines, describe the transformation method, the number of independent lines analyzed and the generation upon which experiments were performed. For gene-edited lines, describe the editor used, the endogenous sequence targeted for editing, the targeting guide RNA sequence (if applicable) and how the editor was applied.*
Authentication	*Describe any authentication procedures for each seed stock used or novel genotype generated. Describe any experiments used to assess the effect of a mutation and, where applicable, how potential secondary effects (e.g. second site T-DNA insertions, mosiacism, off-target gene editing) were examined.*
